# *Platycodon grandiflorum* Extract Alleviates Inflammation During Asthma Development In Vivo and In Vitro

**DOI:** 10.1155/ancp/6617215

**Published:** 2025-10-05

**Authors:** Jie Liu, Wei Li, Jingchao Yu, Jinle Lu, Yanan Wei

**Affiliations:** Department of Head and Neck Thyroid Surgery, Cangzhou Hospital of Integrated TCM-WM, Hebei.Cangzhou City, Hebei 061000, China

**Keywords:** asthma, inflammatory response, *Platycodon grandiflorum*, RTE

## Abstract

**Backgrounds:**

Asthma causes over 1000 deaths daily worldwide and is becoming one of the most prevalent and severe respiratory diseases. This study aimed to investigate the therapeutic effects of *Platycodon grandiflorum* extract (PGE) on asthma both in vivo and in vitro.

**Methods:**

Thirty Sprague–Dawley (SD) rats were divided into named control (NC), asthma, and PGE high-, medium-, and low-dose groups. The PGE groups were intragastrically administered the drug for 14 days. After treatment, pathological changes were observed using histological staining. Flow cytometry was used to observe changes in inflammatory cells. The expression of the toll-like receptor 4/nuclear factor kappa B (TLR4/NF-κB) pathway proteins was detected using western blotting. Additionally, lipopolysaccharide (LPS)-stimulated rat tracheal epithelial (RTE) cells were used for validation in vitro.

**Results:**

Histological staining revealed smooth muscle hyperplasia and inflammatory cell infiltration in asthmatic mice. The pathological condition of the lung tissue of rats in all treatment groups improved, with the high-dose group showing the most significant improvement. PGE treatment reduced the number of inflammatory cells recruited in the lung of asthmatic rats, and reduced tumor necrosis factor alpha (TNF-α), interleukin (IL)-1β, and IL-6 levels. Western blotting showed that TLR4 and p-P65 levels decreased significantly after PGE administration (*p*  < 0.05). Additionally, PGE treatment decreased apoptosis in LPS-stimulated RTE cells and decreased TNF-α, IL-1β, and IL-6 levels.

**Conclusions:**

PGE inhibited inflammatory responses in asthmatic rats, which was validated at the cellular level. This therapeutic mechanism may be achieved through the regulation of the TLR4/NF-κB pathway.

## 1. Introduction

Asthma is a common respiratory disease characterized by airway hyperresponsiveness, inflammation, and remodeling, and has been identified as a global public health concern [1]. Studies have shown that the occurrence and development of asthma are often accompanied by the infiltration of various inflammatory cells, such as eosinophils, macrophages, mast cells, lymphocytes, and airway epithelial cell inflammatory damage, which can further aggravate the body's inflammatory response [2, 3]. Clinical symptoms of asthma include shortness of breath, wheezing, coughing, and chest tightness [4]. Asthma causes more than 1000 deaths per day worldwide and is one of the fastest growing and most serious respiratory diseases [5]. Asthma development involves the overreaction of helper T cell 2 (Th2), resulting in an imbalance in Th1 and Th2 expression and the upregulation of the expression of various cytokines [6]. Therefore, reducing the airway inflammatory response can be used as one of the measures to alleviate bronchial asthma.

The nuclear factor kappa B (NF-κB) pathway is present in most cell types and regulates transcription of a variety of target genes encoding inflammatory proteins under inflammatory conditions [7]. It has been found that NF-κB signaling pathway plays an important role in the pathogenesis of asthma [7, 8]. Growth factors and tissue inhibitors of metalloproteinases, cytokines, and inflammatory mediators involved in this pathway can induce asthma [9]. Studies have shown that cytokine inflammatory transmitters involved in airway inflammation in asthma are regulated by the NF-κB pathway at the gene transcription level [10, 11]. NF-κB is involved in airway inflammation and airway remodeling in asthma [12]. The NF-κB signaling pathway is associated with the development of asthma, and can specifically regulate inflammation, oxidative stress, apoptosis, autophagy, and airway remodeling, thus, affecting the development of asthma, and therefore, is an alternative pathway for the treatment of asthma [7, 13]. Especially in recent years, traditional chinese medicine (TCM) monomers and compounds can target NF-κB signaling pathway to treat asthma, providing a new strategy for the treatment of asthma [14–16].

At present, asthma is treated with glucocorticoids and tracheal relaxants. This treatment temporarily relieves asthma symptoms, but asthma has a long course and is prone to recurrence, so the overall treatment effect is not very satisfactory [1, 2]. TCM has been widely used for the treatment of various diseases, with proven efficacies. *Platycodon grandiflorum* has traditionally been used as a herb and food in China to treat various lung and respiratory diseases [17, 18]. *Platycodon grandiflorum* contains a variety of chemical components, including saponins, polysaccharides, flavonoids, phenolic acids, fatty acids, and sterols [19]. In addition, *Platycodon grandiflorum* has anti-inflammatory and antiallergic pharmacological effects [20]. To develop potential immune enhancers that can be used as functional food ingredients, there is increasing interest in the immunomodulatory properties of plants with a wide range of therapeutic properties, because plant-based therapeutic agents have relatively low toxicities [21]. Therefore, this study aimed to explore the effects of *Platycodon grandiflorum* extract (PGE) treatment in asthmatic rats, focusing on the mediation of the NF-κB pathway.

## 2. Materials and Methods

### 2.1. Preparation of PGE

*Platycodon* decoction was obtained from Zhejiang Chinese Medicine University. Decoction pieces of *P. grandiflorum* were extracted three times using petroleum ether with refluxing at 60–90°C for 4 h per extraction. The petroleum ether was recovered, then three extractions were performed using 80% ethanol reflux for 2 h per extraction to remove the fat soluble and alcohol soluble components. The ethanol was recovered and then extracted three times using distilled water reflux for 2 h per extraction and the filtrate was combined. After concentration, anhydrous ethanol was added and the decoction was allowed to stand overnight. The decoction was then centrifuged, the supernatant was collected, precipitated, and dried. The main components of PGE were analyzed using high-performance liquid chromatography (HPLC). Standards (Platycodin D) for HPLC analysis were obtained from Sigma–Aldrich (St Louis, MO, USA). The dilution (correlation coefficient ≥0.996) of each standard product of platycoside D (1–10 μg/mL) was injected into a HPLC system to establish a calibration curve and quantitatively determine the content of platycoside D in PGE.

### 2.2. Establishment of Animal Model

The rat model of asthma was established according to a previously reported protocol [22]. Thirty male Sprague–Dawley (SD) rats (250 ± 20 g) were purchased from Shanghai Slack Laboratory Animal Co., Ltd. (Shanghai, China). All rats were housed in standard cages with a light/dark cycle of 12 h at a temperature of 18–25°C and relative humidity of 65%–70%. The SD rats were randomly divided into five groups: Healthy control (named control, NC; *n* = 6), asthma model control (asthma, *n* = 6), high-concentration PGE treatment (H-PGE, 500 µg/kg, *n* = 6), medium-concentration PGE treatment (M-PGE, 250 µg/kg, *n* = 6), and low-concentration PGE treatment (L-PGE, 125 µg/kg, *n* = 6) groups according to the published reports [23, 24]. All rats except for those in the NC group were sensitized using intraperitoneal injection of 1 mL of solution containing 100 µg albumin and 200 mg aluminum hydroxide on the 1^st^ and 8^th^ days of the experiment. From the 15^th^ day, the rats were administered atomized inhalation of 1% albumin stimulating solution every day to stimulate the airway for 30 min, once a day for 4 weeks. When the rats showed symptoms such as shortness of breath, irritability, and abdominal muscle twitching, the model was considered successful. The rats in the NC group were sensitized and atomized using normal saline.

### 2.3. PGE Treatment

After modeling, the NC and asthma groups received intragastric administration of normal saline, whereas the remaining treatment groups were intragastrically administered different drug concentrations once daily for 14 continuous days. After treatment, blood samples were obtained from the tail veins of the rats, and serum was collected and stored at −80°C. Subsequently, the rats were euthanized in a CO_2_ chamber. The tissues were dissected to obtain lung tissue sections. Images were obtained using a color pathological image analysis system to evaluate the degree of histopathological changes in the lungs. A portion of the tissue was fixed with 10% neutral-buffered formaldehyde for 24 h to facilitate pathological evaluation and the remaining sections were used for western blotting. The experimental and animal care procedures were approved by Cangzhou Hospital of Integrated TCM-WM (Approval Number. 2020-ky-054.1) and adhered to the guidelines of the National Institutes of Health's Guide for the Care and Use of Laboratory Animals.

### 2.4. Hematoxylin and Eosin Staining

Part of the lung tissue was fixed with 4% paraformaldehyde, dehydrated with alcohol, rendered transparent with xylene, embedded in paraffin, and stone wax sections (5 μm) were prepared. The slices were stained with hematoxylin for approximately 30 min, stained with eosin staining solution for 1 min, rinsed, dehydrated, sealed with transparent neutral gum, and observed under an optical microscope (Leica DM2500M; Leica Microsystems, Wetzlar, Germany).

### 2.5. Detection of Lung Function

The maximum expiratory flow (PEF), maximum intermediate expiratory flow (MMEF) and forced vital capacity (FVC) of rats in each group were measured by small animal lung function detector (Wheat Bang MSA99; Beijing Mai Bang company, Beijing, China) at 24 h after treatment.

### 2.6. Cell Culture and Treatment

It has been shown that lipopolysaccharide (LPS) promoted apoptosis of rat tracheal epithelial (RTE) cells, autophagy, and can be used as an auxiliary cell model for asthma treatment [25]. Therefore, we used RTE cells as a further in vitro validation of the mechanism of action of PGE in asthma treatment. RTE cells lines were purchased from the Cell Bank of the Chinese Academy of Sciences (Shanghai, China) and Beyotime (Hangzhou, China). The vendors confirmed that the cells were identified using STR profiling. All cell lines were examined for the presence of mycoplasma using a LookOut Mycoplasma PCR Detection Kit (Merck & Co., Kenilworth, NJ, USA). All cells were cultured at 37°C with 5% CO_2_ in minimal essential medium containing 20% fetal bovine serum (Gibco, Rockville, MD, USA) and subcultured every 2–3 days for subsequent experiments. Blood samples from the abdominal aorta were collected from normal rats and rats in the PGE low-, medium-, and high-dose groups. The samples were allowed to stand at room temperature for 30 min, then centrifuged at 3000 × *g* for 20 min. The serum was incubated in a 56°C water bath for 30 min and filtered and sterilized using a 0.22 µm filter.

To further explore the antiasthmatic mechanism of PGE, RTE cells were randomly divided into five groups. Five groups of cells were separately cultured in a medium containing blank rat serum or medium containing serum from rats supplemented with LPS or PGE (low, medium, and high doses), and NC, LPS, H-PGE, M-PGE, and low-concentration PGE treatment (L-PGE). After 24 h of treatment, cells were harvested for flow cytometry, western blot analysis, Enzyme-Linked Immunosorbent Assay (ELISA), and quantitative reverse transcription polymerase chain reaction (qRT-PCR), respectively.

### 2.7. qRT-PCR

Total RNA was extracted from the collected lung tissues and treated RTE cells using TRIzol reagent according to the manufacturer's instructions. Amplification was performed using a SYBR green PCR master mix kit (Thermo Fisher, Waltham, MA, USA) on a 7900HT fast real-time system (Thermo Fisher). The relative expression of proteins was calculated using the 2^−ΔΔCt^ method, and U6 was used as an endogenous control. The primers used in this study are listed in Table 1.

### 2.8. Flow Cytometry

Flow cytometry was used to measure the number of inflammatory cells (neutrophils, T lymphocytes, and macrophages) in lung tissue. The lung tissue was chopped and digested with collagenase IV to obtain a dissociated cell suspension. After staining the cell particles with PE-labeled anti-CD11B, APC-labeled anti-GR-1, fluorescein isothiocyanate (FITC)-labeled anti-CD3, PerCP-labeled anti-CD45, and APC-labeled anti-F4/80 antibodies, the cells were fixed with 4% formaldehyde and the number of inflammatory cells was calculated using flow cytometry. Additional, bronohoalveolar lavage fluid was collected from rats and centrifuged at 350 × *g* for 5 min at 4°C. Supernatants were to detect the changes in neutrophils, eosinophils, and T lymphocytes.

### 2.9. Apoptosis Analysis

An annexin V-FITC binding assay was used to quantify the percentage of early and late apoptotic cells. The cells (2 × 10^5^ cells/well) were seeded into 24-well plates and incubated for 24 h. The cells were then treated with the optimum concentration of PGE for 24 h. Subsequently, adherent and nonadherent cells were harvested prior to staining with annexin V-FITC and propidium iodide (PI), according to the manufacturer's instructions.

### 2.10. Enzyme-Linked Immunosorbent Assays (ELISAs)

Rat serum and cell supernatants were collected after treatment. The levels of interleukin (IL)-1β, IL-6, and tumor necrosis factor alpha (TNF-α) were measured following the manufacturer's instructions (Nanjing Jiancheng Bioengineering Institute, Nanjing, China). The absorbance of each well was measured at 450 nm.

### 2.11. Western Blot

Proteins from tissues or cells were extracted and separated using 10% sodium dodecyl sulfate-polyacrylamide gel electrophoresis. The protein concentrations in the supernatants were quantified using a micro BCA protein assay kit (Pierce, Rockford, IL, USA). Equal amounts (20 µL) of protein were loaded onto the gels, and the separated proteins were transferred onto polyvinylidene fluoride membranes. The membranes were blocked in tris-buffered saline containing 5% Tween 20% and 5% skim milk at room temperature for 1 h and incubated overnight at 4°C using a primary antibody: Toll-like receptor 4 (TLR4), phospho-NF-κB p65 (p-P65), P65, and GAPDH (1:1000; all antibodies were purchased from Cell Signaling Technology, Boston, MA, USA). Primary antibodies were detected using a secondary antimouse or antirabbit IgG antibody coupled with horseradish peroxidase (HRP; 1:5000, ab6728, Abcam, Cambridge, UK). Target proteins were visualized using an EZ-ECL chemiluminescence detection kit (Pierce, Rockford, IL, USA). The relative density was analyzed on a Molecular imager ChemiDoc XRS system (Bio-Rad Laboratories, Hercules, CA) using enhanced chemiluminescence reagent (Thermo Fisher Scientific, Shanghai, China).

### 2.12. Statistical Analysis

Data were analyzed using the GraphPad Prism 8.0 software (GraphPad Software, Boston, MA, USA). Results are presented as the mean ± standard deviation. Student's *t*-test was performed for comparison between two groups. Differences among multiple groups were assessed using one-way analysis of variance (ANOVA) with Tukey's post hoc analysis. Statistical significance was set at *p* < 0.05.

## 3. Results

### 3.1. HPLC Analysis of PGE

**Figure 1:**
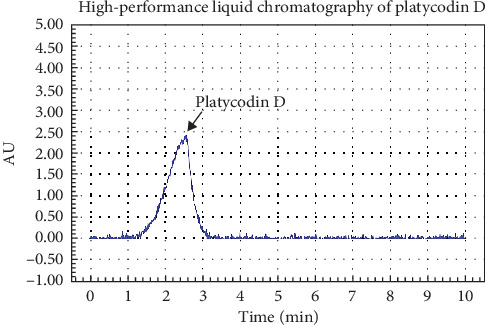
Representative high-performance liquid chromatography profile of PGE. Platycodin D was detected by LC–MS/MS.

The physiological activity of platycodon is mainly derived from its active ingredient platycodon D (Figure 1). After analysis of platycodin, the results showed that the main content of platycodin D was 23.5 mg/L.

### 3.2. PGE Alleviates Inflammatory Response in Asthmatic Model Rats

**Figure 2:**
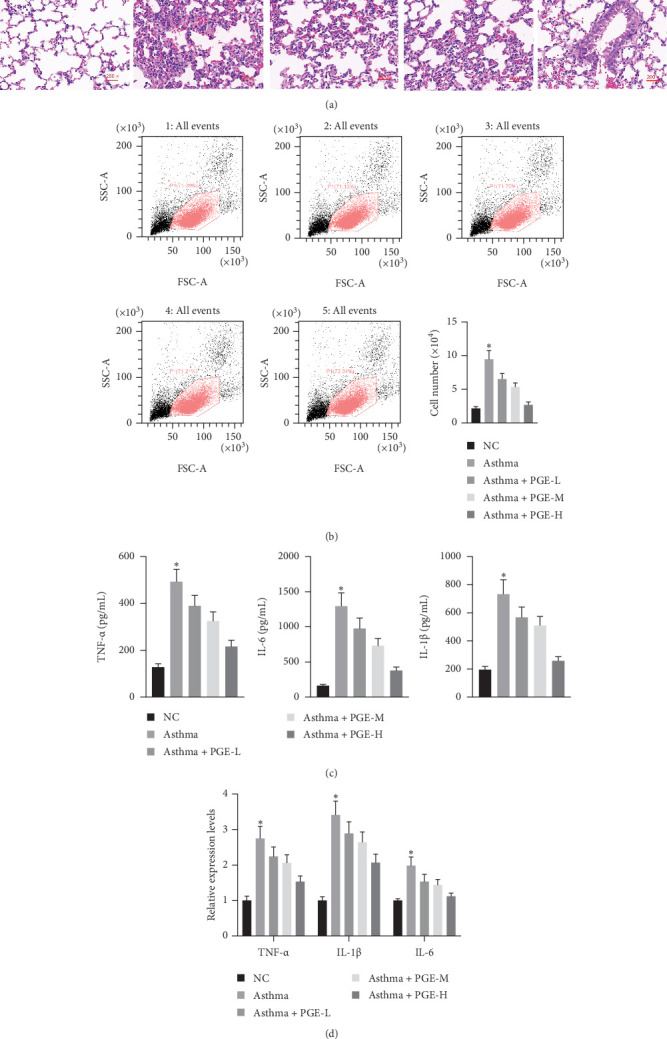
PGE alleviates inflammatory response in asthmatic model rats. (A) Hematoxylin and eosin staining of each group at 200 × magnification. (B) Flow cytometry to detect inflammatory cell recruitment. (C) ELISA was used to determine changes of serum cytokines in each group. (D) qRT-PCR was used to determine changes of serum cytokines in each group. *n* = 6, *⁣*^*∗*^*p* < 0.05.

We first established a rat model of asthma. No abnormal changes were observed in the NC group; the lung tissue was normal in shape and clear in structure, with no inflammatory cell infiltration or abnormalities observed in the bronchus and peripheral blood vessels. However, in the asthmatic model group, the structures of the alveoli and bronchi were disordered, and a large number of infiltrated inflammatory cells and mucous plugs were observed (Figure 2A). We also observed protective effects of different doses of PGE against asthma. The findings revealed that, in the treatment groups, the lung tissue structure and morphology were improved, inflammatory cell infiltration and submucosal edema were reduced, and airway stenosis was less common. Among them, this improvement was most evident in the high-dose group (Figure 2A). Flow cytometry showed that the total number of inflammatory cells in the asthma group was significantly higher than that in the NC group. Different doses of PGE reduced the total number of inflammatory cells recruited to the lungs, especially in the high-dose group (Figure 2B and Table 2). As shown in Figure 2C, D, the expression of TNF-α, IL-1β, and IL-6 increased in the asthma group. However, the levels of TNF-α, IL-1β, and IL-6 were partially decreased after PGE treatment, and the decrease was significant in the high-dose group. Additional, compared with NC group, MMEF, PEF, and FVC of asthmatic rats were significantly decreased (*p* < 0.05; Table 3).

### 3.3. PGE Alleviates Inflammatory Response of LPS-Stimulated Rat RTE Cells

**Figure 3:**
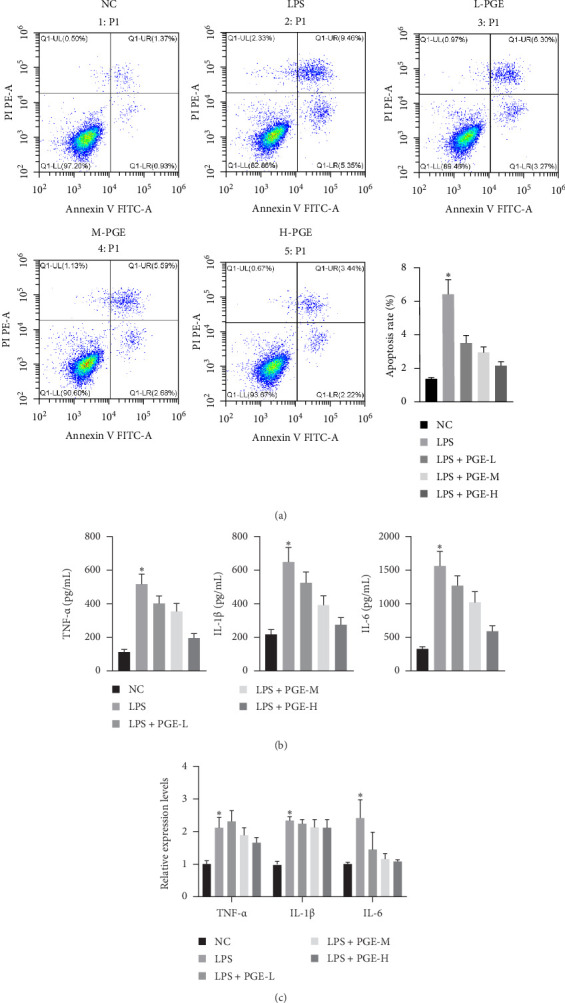
PGE alleviates inflammatory response of LPS-stimulated rat RTE cells. (A) Flow cytometry to detect apoptosis. (B) ELISA was used to determine changes of serum cytokines in each group. (C) qRT-PCR was used to determine changes of serum cytokines in each group. *⁣*^*∗*^*p* < 0.05.

Subsequently, we examined the effects of PGE on the apoptosis of LPS-stimulated RTE cells. Flow cytometry showed that the apoptosis of cells treated with different doses of PGE was significantly lower than that of the LPS group, especially in the high-dose group (Figure 3A and Table 4), indicating that PGE inhibited LPS-mediated apoptosis. In addition, compared with the NC group, the levels of TNF-α, IL-1β, and IL-6 were increased in the LPS group. However, after PGE treatment, the levels of TNF-α, IL-1β, and IFN-γ in the cells were partially reduced, especially in the high-dose group (Figure 3B, C).

### 3.4. PGE Reduced the Expression of NF-κB Signaling Pathway Components

**Figure 4:**
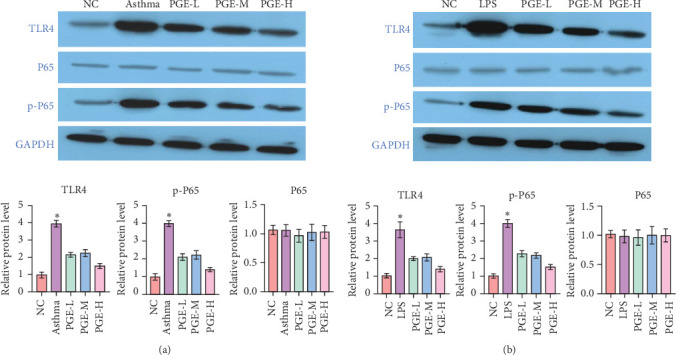
Expression of the NF-κB signaling pathway components in vivo and in vitro. (A) Western blot analysis showing NF-κB signaling pathway protein expression levels in asthma model rats. *n* = 6. (B) Western blot analysis showing NF-κB signaling pathway protein expression levels in RTE cells. *⁣*^*∗*^*p* < 0.05.

TLR4 and p-P65 levels in the asthma group increased significantly compared to those in the control group, whereas these expression levels decreased in asthmatic rats treated with PGE (Figure 4A). This effect became more pronounced with increasing PGE concentrations. In addition, the results in LPS-stimulated RTE cells were consistent with those in the in vivo experiment (Figure 4B).

## 4. Discussion

Asthma is a chronic airway inflammatory disease involving eosinophils, neutrophils, airway epithelial cells, and other cell components [26]. Inflammatory effector cells, primarily eosinophils, mediate airway tissue inflammation [27]. The secretion of a large number of cytokines may cause inflammatory cells, such as eosinophils, to become chemotactic. This results in inflammatory cells infiltrating the trachea and airway mucosa, airway smooth muscle contraction, and eventual asthma development [28]. Airway inflammation is caused by a Th2 overreaction, which leads to an imbalance in Th1 expression [29]. Studies have shown that PGE has the potential to treat asthma due to its anti-inflammatory effects [30]. However, the evaluation of the anti-inflammatory effects in vitro and in vivo for asthma treatment has not been reported. In the present study, a rat model of asthma was successfully established. The results showed that pulmonary histological changes, bronchiolar wall inflammatory cell infiltration, airway goblet cell proliferation, and mucus secretion increased in the asthma group. PGE effectively reduced lung inflammation [31]. Flow cytometry also revealed a decrease in the number of inflammatory cells in asthmatic rats after PGE treatment.

TNF-α, IL-6, and IFN-γ have been reported to mediate airway inflammation in asthma [32]. The results of this study showed that TNF-α, IL-6, and IFN-γ were significantly increased in asthmatic rats, but the levels of these inflammatory factors could be reduced after PGE treatment, demonstrating the anti-inflammatory effect of PGE in asthma. To further demonstrate the anti-inflammatory effects of PGE in the treatment of asthma at the cellular level, we cultured LPS-stimulated RTE cells using the serum from PGE-treated asthmatic rats. This study found that the levels of TNF-α, IL-6, and IFN-γ in LPS-stimulated RTE cells were reduced after treatment, and the rate of apoptosis was reduced, indicating the efficacy of PGE for asthma treatment at the cellular level.

The TLR4/NF-κB pathway is a classic inflammatory signaling pathway [33]. NF-κB is present in most cell types and, under inflammatory conditions, NF-κB translocates from the cytoplasm to the nucleus to regulate the expression of various target genes encoding inflammatory proteins [34]. Iκb is a specific inhibitor of NF-κB, so the inactivated NF-κBp65 and iκB are the forms of NF-κB present in the cytoplasm at rest, and when the cell is triggered by an exogenous signal, IκB releases NF-κB into the nucleus to regulate gene expression, and the NF-κB signaling pathway is activated [35]. It has been reported that cytokine inflammatory transmitters involved in airway inflammation in asthma are regulated by NF-κB pathway at the gene transcription level, and NF-κB is involved in airway inflammation and airway remodeling in asthma. Inhibition of the NF-κB pathway can effectively reduce airway inflammation, airway remodeling, and hypersecretion of mucus [36]. However, the NF-κB pathway still has limitations in the treatment of asthma. The NF-κB pathway is in a complex signaling network during the course of asthma and does not exist independently. The TGF-β1/Smad, MAPK, and PI3K/AKT/mTOR pathways also play roles in the treatment of asthma [37]. Therefore, the interaction relationships among these pathways need to be further evaluated.

Studies have shown that PGE has a significant effect on allergic and respiratory diseases, and its effect is mainly related to the anti-inflammatory function [31]. In this study, the expression of TLR4 and p65 proteins in the lung tissue and LPS-stimulated PTE cells of asthmatic rats was upregulated by PGE treatment. This indicates that the reduction of airway inflammation by PGE may be related to the inhibition of the TLR4/NF-κB pathway. However, this mechanism needs to be verified through further experiments.

This study has some limitations. An increase in the therapeutic concentration of PGE resulted in a stronger inhibitory effect on asthma. However, the optimal therapeutic concentration of PGE and which components of PGE play key therapeutic roles need to be determined in future studies. In addition, this study found that PGE has an effect on the expression of NF-κB pathway during treatment; however, whether the mechanism of action of PGE therapy is related to NF-κB pathway needs more comprehensive experimental evidence. Moreover, in the rat sex selection, we selected all males, although this selection is common, but our future research also needs to evaluate whether there is a difference in the effect of PGE in females and males. Furthermore, more detailed histological assessments will be conducted in future experiments, such as assessing lung injury by quantifying mucus-secreting goblet cells based on periodic acid Schiff (PAS) staining, to more fully evaluate the therapeutic effect of PGE. And the changes of inflammatory markers in BALF need to be evaluated in future studies. Finally, the therapeutic mechanism of PGE on inflammatory cells and RTE cells will be analyzed in more detail in future experiments.

## 5. Conclusion

PGE can inhibit inflammation during the development of asthma, and its mechanism may involve the regulation of the NF-κB pathway. These results provide new therapeutic options for the treatment of asthma.

## Figures and Tables

**Table 1:** Primer information in this study.

Primer name	Sequences
IL-1β forward	5′-GGTTCAAGGCATAACAGGCTC-3′
IL-1β reverse	5′-TCTGGACAGCCCAAGTCAAG-3′
TNF-α forward	5′-GGTGACCTTGTGTGTGCTTG-3′
TNF-α reverse	5′-ATGTCCTGAGCCATGGAAGC-3′
IL-6 forward	5′-GGTGACCTTGTGTGTGCTTG-3′
IL-6 reverse	5′-ATGTCCTGAGCCATGGAAGC-3′
U6 forward	5′-GCTTCGGCAGCACATATACTAAAAT-3′
U6 reverse	5′-CGCTTCACGAATTTGCGTGTCAT-3′

**Table 2:** The number of various inflammatory cells in BALF (*n* = 6).

Group	Eos (×10^4^)	Mac (×10^4^)	Lym (×10^4^)	Neu (×10^4^)	Tatal (×10^4^)
NC	0.16 ± 0.08	1.09 ± 0.31	0.86 ± 0.16	0.14 ± 0.04	2.15 ± 0.27
Asthma	1.85 ± 0.58*⁣*^*∗*^	4.16 ± 0.91*⁣*^*∗*^	2.81 ± 0.54*⁣*^*∗*^	0.64 ± 0.22*⁣*^*∗*^	9.46 ± 1.31*⁣*^*∗*^
L-PGE	1.36 ± 0.56	3.35 ± 0.56	1.67 ± 0.24*⁣*^*∗∗*^	0.54 ± 0.21	6.52 ± 0.84*⁣*^*∗∗*^
M-PGE	0.92 ± 0.46*⁣*^*∗∗*^	2.56 ± 0.25*⁣*^*∗∗*^	1.16 ± 0.38*⁣*^*∗∗*^	0.39 ± 0.16*⁣*^*∗∗*^	5.33 ± 0.61*⁣*^*∗∗*^
H-PGE	0.47 ± 0.25*⁣*^*∗∗*^	1.34 ± 0.65*⁣*^*∗∗*^	0.62 ± 0.31*⁣*^*∗∗*^	0.24 ± 0.09*⁣*^*∗∗*^	2.67 ± 0.43*⁣*^*∗∗*^

Abbreviations: Eos, eosinophil; Lym, lymphocyte; Mac, macrophage; Neu, neutrophil.

*⁣* ^*∗*^Compared with NC group, *p* < 0.05.

*⁣* ^*∗∗*^Compared with asthma group, *p* < 0.05.

**Table 3:** Lung function PEF, MMEF, and FVC in rats (*n* = 6).

Group	PEF (L/s)	MMEF (L/s)	FVC (L)
NC	4.86 ± 0.57	9.45 ± 1.65	3.45 ± 0.57
Asthma	1.59 ± 0.33*⁣*^*∗*^	2.46 ± 0.34*⁣*^*∗*^	1.16 ± 0.67*⁣*^*∗*^
L-PGE	2.16 ± 0.36	3.66 ± 0.82	1.79 ± 0.36*⁣*^*∗∗*^
M-PGE	2.92 ± 0.51*⁣*^*∗∗*^	5.82 ± 0.41*⁣*^*∗∗*^	2.34 ± 0.41*⁣*^*∗∗*^
H-PGE	3.83 ± 0.67*⁣*^*∗∗*^	7.68 ± 0.74*⁣*^*∗∗*^	3.04 ± 0.63*⁣*^*∗∗*^

Abbreviations: FVC, forced vital capacity; MMEF, maximum intermediate expiratory flow; PEF, maximum expiratory flow.

*⁣* ^*∗*^Compared with NC group, *p* < 0.05.

*⁣* ^*∗∗*^Compared with asthma group, *p* < 0.05.

**Table 4:** Apoptosis rates of cells in each group (*n* = 6).

Group	Apoptosis rate (%)
NC	1.38 ± 0.09
Asthma	6.42 ± 0.87*⁣*^*∗*^
L-PGE	3.51 ± 0.46*⁣*^*∗∗*^
M-PGE	2.95 ± 0.33*⁣*^*∗∗*^
H-PGE	2.16 ± 0.25*⁣*^*∗∗*^

*⁣* ^*∗*^Compared with NC group, *p* < 0.05.

*⁣* ^*∗∗*^Compared with asthma group, *p* < 0.05.

## Data Availability

The data that support the findings of this study are available from the corresponding author upon reasonable request. The data are not publicly available due to privacy or ethical restrictions.
